# Identification and Characterization of Cancer Stem-Like Cells in ALK-Positive Anaplastic Large Cell Lymphoma Using the SORE6 Reporter

**DOI:** 10.3390/cimb43020041

**Published:** 2021-07-02

**Authors:** Jing Li, Moinul Haque, Chuquan Shang, Bardes Hassan, Dongzhe Liu, Will Chen, Raymond Lai

**Affiliations:** 1 Department of Laboratory Medicine and Pathology, University of Alberta, Edmonton, AB T6G 2E1, Canada; lijing2020@hrbmu.edu.cn (J.L.); moinul@ualberta.ca (M.H.); chuquan@ualberta.ca (C.S.); dr_bardis@cu.edu.eg (B.H.); dongzheliu@szu.edu.cn (D.L.); will.chen@ualberta.ca (W.C.); 2 Electron Microscopy Center, Basic Medical Science College, Harbin Medical University, Harbin 150080, China; 3 College of Medicine and Health, University College Cork, T12 AK54 Cork, Ireland; 4 Department of Pathology, Faculty of Veterinary Medicine, Cairo University, Giza 12211, Egypt; 5 Laboratory of Biology and Chemistry, Basic Medical Science College, Harbin Medical University, Harbin 150080, China; 6 Department of Oncology, University of Alberta, Edmonton, AB T6G 2R7, Canada

**Keywords:** anaplastic large cell lymphoma, SORE6, cancer stem-like cells, Sox2, Oct4

## Abstract

Transcription factors Sox2 and Oct4 are essential in maintaining the pluripotency of embryonic stem cells and conferring stemness in cancer stem-like (CSL) cells. SORE6, an in-vitro reporter system, was designed to quantify the transcription activity of Sox2/Oct4 and identify CSL cells in non-hematologic cancers. Using SORE6, we identified and enriched CSL cells in ALK-positive anaplastic large cell lymphoma (ALK + ALCL). Two ALK + ALCL cell lines, SupM2 and UCONN-L2, contained approximately 20% of SORE6+ cells, which were purified based on their expression of green fluorescent protein. We then performed functional studies using single-cell clones derived from SORE6− and SORE6+ cells. Compared to SORE6− cells, SORE6+ cells were significantly more chemoresistant and clonogenic in colony-formation assays. Sox2/Oct4 are directly involved in conferring these CSL properties, since the shRNA knockdown of Sox2 in SORE6+ significantly lowered their chemoresistance, while enforced expression of Sox2/Oct4 in SORE6− cells produced opposite effects. Using Western blots, we found that the expression and subcellular localization of Sox2/Oct4 were similar between SORE6− and SORE6+ cells. However, in SORE6+ but not SORE6− cells, Sox2 and Oct4 abundantly bound to a probe containing the SORE6 consensus sequence. c-Myc, previously shown to regulate cancer stemness in ALK + ALCL, regulated the SORE6 activity. In conclusion, SORE6 is useful in identifying/enriching CSL cells in ALK + ALCL.

## 1. Introduction

ALK-positive anaplastic large cell lymphoma (ALK + ALCL) is a distinct type of non-Hodgkin lymphoma of T-cell lineage occurring most frequently in young adults and children [1,2,3,4]. A key molecular signature of ALK + ALCL is the reciprocal chromosomal translocation, the t(2:5)(p23;35) abnormality [5]. This results in the generation of the oncogenic fusion protein termed NPM-ALK, as an aberrant fusion of nucleophosmin (NPM1) and anaplastic lymphoma kinase (ALK) [6]. NPM-ALK is a functionally versatile oncoprotein, and the mechanisms by which it promotes tumorigenesis has been extensively studied and reviewed [7]. Clinically, while the overall prognosis associated with ALK + ALCL is better than that of other types of T-cell malignancies, disease relapses following conventional treatments are relatively frequent [8,9,10]. Recent studies in cancer biology have postulated that cancer relapses might be attributed to the existence of a small subpopulation of cancer stem cells that are characterized by high levels of chemoresistance, self-renewal, and pluripotency [11,12]. In this model, the majority of tumors are typically eradicated by most traditional cancer treatments, but the cancer stem cells persist and provide the seeds for disease relapses, at which time tumors will become more chemoresistant than those found at the initial presentation [13,14,15].

The identification and enrichment of cancer stem cells have been described in various cancer models, and the methods most commonly involve the detection of specific cell-surface markers (e.g., CD133, CD44, CD24) and cytoplasmic proteins (e.g., aldehyde dehydrogenase, ALDH1) [16,17,18,19]. In the literature, researchers have also reported the existence of cells, often labeled as cancer stem-like (CSL) cells, which possess some but not all of the properties of cancer stem cells. One approach to detect CSL cells is based on the expression and transcriptional activity of inducible pluripotent stem cell (iPS) proteins such as Sox2 and Oct4, which normally play a key role in maintaining the pluripotency of embryonic stem cells [20,21]. While the expression of Sox2 and Oct4 is normally restricted to embryonic stem cells, the aberrant expression of these two proteins has been found in various types of cancer. Our group has previously shown that Sox2 expression can be induced by the NPM-ALK/STAT3 axis in ALK + ALCL cells, and a small subset of cells showing Sox2 transcriptional activity detectable by using a Sox2 reporter is enriched with CSL cells [22]. However, the expression and transcriptional activity of Oct4 in ALK + ALCL have not been examined. In 2015, a novel reporter labeled SORE6 designed to measure the transcriptional activity of both Sox2 and Oct4 was generated [20]. Specifically, SORE6 consists of six tandem repeats of a composite Sox2/Oct4 response element derived from the promoter of the human *NANOG* gene [20]. This system has been tested in a small number of non-hematologic malignancies including pleomorphic sarcoma [23], gastric cancer [24], breast cancer [20], and malignant mesothelioma [25]. Compared to SORE6-negative (SORE6−) cells, SORE6-positive (SORE6+) cells were found to be significantly more chemoresistant and capable of self-renewal, tumor initiation, and asymmetric cell division. Whether SORE6 is a useful tool to identify and enrich CSL cells in hematologic malignancies such as ALK + ALCL has not been previously tested.

In our study, we aim to assess whether SORE6 is a useful tool to facilitate the detection and enrichment of CSL cells in ALK + ALCL. Thus, using a lentiviral vector carrying the SORE6 reporter and two ALK + ALCL cell lines, we generated and purified single-cell SORE6+ and SORE6− cell clones. Results from our functional studies support the concept that the SORE6 reporter is useful in identifying and enriching CSL cells in ALK + ALCL. In view of our previous finding that c-Myc (another embryonic stem cell protein) can regulate cancer stemness in ALK + ALCL [26], we also asked if the expression level of c-Myc can regulate the SORE6 activity in these cells.

## 2. Materials and Methods

### 2.1. Culture of Cell Lines

Five ALK + ALCL cell lines were used in this study, including SupM2, UCONN-L2, Karpas 299, SUDHL-1, and SR2. These cells were cultured in RPMI 1640 (Invitrogen, Burlington, ON, Canada) supplemented with 10% fetal bovine serum (Invitrogen, Burlington, ON, Canada) as well as 1% penicillin and streptomycin (Invitrogen). The human teratocarcinoma cell line Ntera-2 was cultured in Dulbecco’s modified Eagle’s medium (Gibco, Carlsbad, CA, USA) containing 10% fetal bovine serum and 1% penicillin and streptomycin. All of the cells mentioned above were purchased from American Type Culture Collection (Manassas, VA, USA). Peripheral blood mononuclear cells were obtained from healthy individuals and isolated using Ficoll-Paque (GE Healthcare Bio-Sciences Crop., Piscataway, NJ, USA)

### 2.2. Antibodies and Drugs

The primary antibodies used were anti-c-Myc (Y69, ab32072) and anti-Oct4 (1:500, ab19857), obtained from Abcam (Abcam, Cambridge, MA, USA). Anti-β-actin (1:3000, sc-47778), anti-α-tubulin (1:2000, c-5286), anti-HDAC-1 (1:500, SC, #81598), and anti-α-actinin (1:500, SC, 17829) were purchased from Santa Cruz Biotechnology (Santa Cruz, CA, USA). All of the following antibodies were purchased from Cell Signaling (Danvers, MA, USA): anti-Sox2 (1:1000, D6D9), anti-KLF4 (1:500, CST, #4038S), anti-STAT3 (1:1000, CST, #124H6), anti-pSTAT3^Y705^ (1:2000, CST, #9145), anti-pALKY1604 (1:500, CST, #3341S), and anti-ALK (1:1000, CST, #3633). The secondary antibodies used were HRP-conjugated anti-mouse (1:2000, CST, #7076) and anti-rabbit (1:2000, CST, #7074). Puromycin, doxorubicin, crizotinib, and etoposide were all purchased from Sigma-Aldrich (Oakville, ON, Canada). All treatments were performed following the manufacturer’s instructions.

### 2.3. Vectors and Plasmids

The lentiviral reporter construct SORE6-mCMVp-dsCopGFP-PURO and negative control plasmid mCMVp-dsCopGFP-PURO were kind gifts from Dr. Lalage Wakefield (National Cancer Institute, NIH, Bethesda, MD, USA) [20]. Short hairpin RNA (shRNA) for *Oct4* (TRCN0000004881) was purchased from Sigma. The Oct4 expression vector (pLV-EF1a-hOCT4-IRES-Neo) was purchased from BiOSETTIA (San Diego, CA, USA). shRNA for *Sox2* (#26353) and the *Sox2* expression vector (#16577) were purchased from Addgene (Watertown, MA, USA).

### 2.4. Generation of SORE6+/SORE6− Cells and Flow Cytometry

Lentiviral particles were generated by transfecting the 293T packaging cells (Clontech Laboratories, Inc., Mountain View, CA, USA) with the SORE6-mCMVp-dsCopGFP-PURO and negative control plasmid mCMVp-dsCopGFP-PURO. Two ALK + ALCL cell lines, SupM2 and UCONN-L2, were transduced with the generated viral supernatant in the presence of polybrene twice, 24 h apart. At 24 h after the second lentiviral transduction, cells were cultured in the presence of 0.5 μg/mL puromycin (Sigma-Aldrich, St. Louis, MO, USA). Using flow-cytometric single-cell sorting, we generated multiple single-cell clones derived from SORE6− and SORE6+ cell subsets. For all flow cytometry experiments, the parental SupM2 cell line was used to gate GFP staining cells. Fold change was determined by dividing the experimental group with the control negatively stained parental cells.

### 2.5. Colony Formation in Soft Agar

The soft agar consisted of two layers, both of which were prepared from agar (Invitrogen) dissolved in distilled water and autoclaved. For the bottom layer, 2× RPMI1640 medium supplemented with 20% FBS was added to the 1% agar. For the top layer, cell suspension (6000–7000 cells/mL) in RPMI1640 medium supplemented with 20% FBS was mixed with 0.6% agar. Colonies were stained and visualized with 0.05% crystal violet after 10 days of culture.

### 2.6. Cell Viability Assay

Cell viability experiments were performed in 96-well plates at an initial concentration of 10,000 cells/well with 5% fetal bovine serum. Cell viability was determined using trypan blue exclusion and the 3-(4,5-dimethylthiazol-2-yl)-5-(3-carboxymethoxyphenyl)-2-(4-sulfophenyl)-2H-tetrazolium, inner salt (MTS) assay (Promega, Madison, WI, USA) according to the manufacturer’s protocol.

### 2.7. SORE6 Probe Binding Assay

Cells were harvested and washed with cold PBS, following by cytoplasmic and nuclear fractionation using the Pierce NE-PER kit (Fisher Scientific Canada, Ottawa, ON, Canada). Nuclear proteins (400 µg) were incubated with 3 pmol of oligomers possessing the SORE6 sequence with or without a biotinylated tag (purchased from IDT, Edmonton, AB, Canada). The mixture was rotated at room temperature for 30 min. Streptavidin agarose beads (50 μL, Fisher Scientific) were added to each sample, followed by overnight rotation at 4 °C. The next day, the samples were washed with cold PBS 4 times, and protein was eluted at 100 °C in 4× protein loading buffer for 5 min, followed by Western blotting. The sequence of the duplex SORE6 probe is as follows: 5′-BiosgCCCTTTTGCATTACAATGTCTTTTGCATTACAATGTCTTTTGCATTACAATG-3′.

### 2.8. Reverse Transcription-Polymerase Chain Reaction (RT-PCR)

Total RNA was extracted from cell lines with the RNase Plus Mini Kit (Qiagen, Valencia, CA, USA). Reverse transcription (RT) reactions were performed with 1 μg of total RNA using the Superscript First-Strand Synthesis System Kits (Invitrogen, Carlsbad, CA, USA). RT-PCR parameters included an initial denaturing step at 95 °C for 10 min followed by 30 cycles at 95 °C for 45 s, annealing at 56 °C for 30 s, and extension at 72 °C for 30 s, followed by a final extension step at 72 °C for 10 min. PCR products were then analyzed by agarose gel electrophoresis. Genomic DNA was extract from cell lines with DNeasy Blood & Tissue Kit. Forward (F) and reverse (R) primer sequences: *Sox2* (F:5′-GCCGAGTGGAAACTTTTGTCG-3′; R: 5′-GGCAGCGTGTACTTATCCTTCT-3′), *Oct4* (5′-CTTCTCGCCCCCTCCAGGT-3′; R:5′-AAATAGAACCCCCAGGGTGAGC-3′), *SORE6* (5′-ACAATGGCCTTGGTGCAG-3′; R:5′-TGCACCAAGGCCATTGTAA-3′), and *GAPDH* (5′-GGAGCGAGATCCCTCCAAAAT-3′; R: 5′-GGCTGTTGTCATACTTCTCATGG-3′).

### 2.9. Statistical Analysis

All statistical analyses were performed with GraphPad Prism 7 (Graphpad Software Inc, LaJolla, CA, USA). Flow-cytometric analysis was performed with FlowJo (Ashland, OR, USA). The two-tailed Student’s *t*-test was used to calculate *p*-values. Results are presented as mean ± standard error of the mean.

## 3. Results

### 3.1. Expression of Sox2 and Oct4 in ALK + ALCL Cells

**Figure 1:**
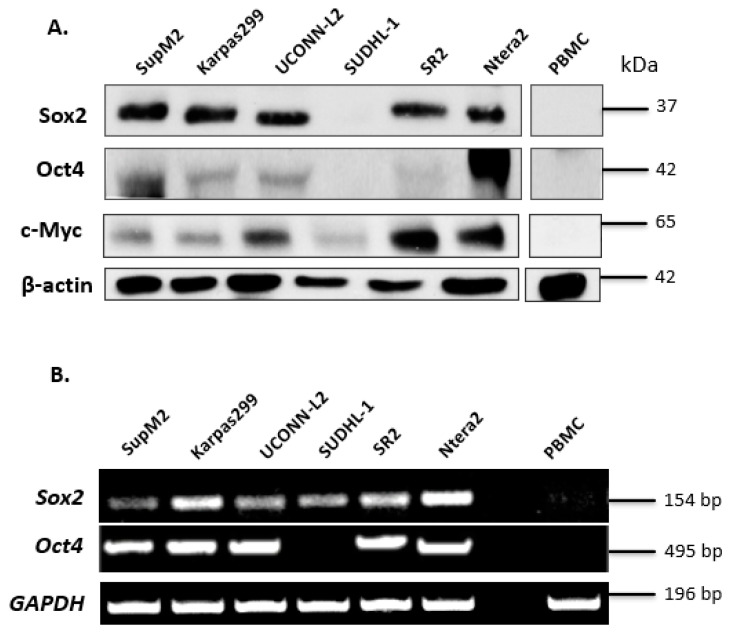
Expression of iPS factors in ALK + ALCL cells. (**A**) Western blotting analysis of the iPS factors (Sox2, Oct,4 and c-Myc) in a panel of 5 ALK + ALCL cell lines. Ntera-2 cells were used as a positive control for Oct4. PBMC was used as a negative control. (**B**) *Sox2* and *Oct4* mRNA expression in five ALK + ALCL cell lines; Ntera-2 and PBMC were measured by PCR.

Since the SORE6 reporter was originally designed to detect the transcription activity of Sox2 and Oct4, two of the four originally described iPS factors, we examined the protein expression of these two proteins in a cohort of five ALK + ALCL cell lines including SupM2, Karpas 299, UCONN-L2, SU-DHL-1, and SR2. We also included c-Myc in our current studies, because our group has previously shown that this protein, which is another iPS factor, can cross-activate the Sox2 reporter (i.e., SRR2) [22]. Ntera-2, a human embryonic carcinoma cell line [27], served as a positive control. Peripheral blood mononuclear cells (PBMCs) from a healthy individual were used as a negative control. As shown in Figure 1A, with the exception of SU-DHL-1, Sox2 was readily detectable in all ALK + ALCL cell lines at 35 kDa. Compared to Ntera-2 cells, ALK + ALCL cells expressed a relatively low level of Oct4, with SupM2 being the only exception. We also found that c-Myc was readily detectable in all ALK + ALCL cell lines, with UCONN-L2 and SR2 being the highest expressors.

To substantiate our Western blot findings, we examined the mRNA levels of *Sox2* and *Oct4* in ALK + ALCL cell lines. As shown in (Figure 1B), with the exception of SU-DHL-1, the mRNA species of both *Sox2* and *Oct4* were identified in all five ALK + ALCL cell lines tested. The absence of detectable *Oct4* mRNA in SU-DHL-1 correlates with its lack of detectable protein expression. The presence of detectable *Sox2* mRNA in SU-DHL-1 suggests that its lack of detectable Sox2 protein expression is due to ineffective translation and/or rapid protein degradation. Ntera-2 and PBMC served as the positive and negative control, respectively.

### 3.2. SORE6 Activity Is Found in a Small Subset of ALK + ALCL Cells

**Figure 2:**
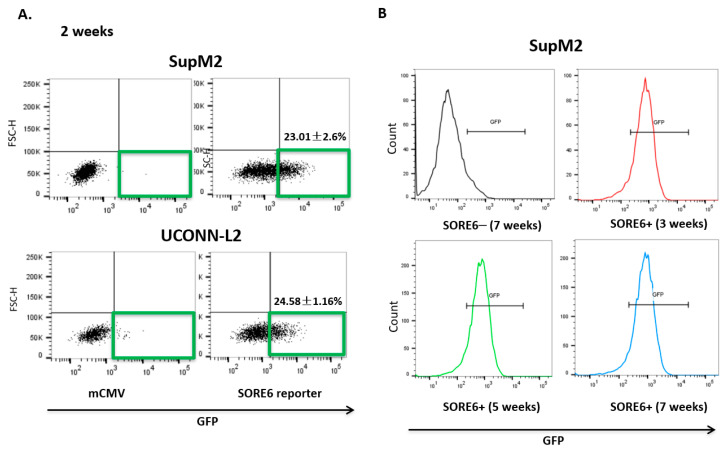
SORE6 reporter is transcriptionally active in relatively small subsets of ALK + ALCL cells. (**A**) mCMV and SORE6 reporters were introduced to two ALK + ALCL cell lines, SUPM2 and UCONN-L2. The gated region shows the proportion of cells that were positive for green fluorescent protein (GFP) expression, both SupM2 and UCONN-L2 cells were detected by flow cytometry after puromycin selection 7 days. (**B**) Analysis of GFP levels in SupM2 SORE6+ cells over the course for 3, 5, and 7 weeks by flow cytometry. SupM2 SORE6− cells at 7 weeks were included as a gating control.

Upon lentiviral transduction of the SORE6 reporter, both SupM2 and UCONN-L2 showed around 20% of SORE6+ cells, detectable by flow cytometry based on their expression of green fluorescent protein (GFP) (Figure 2A). We then purified SORE6− and SORE6+ cells based on their differential GFP expression using a flow-cytometric cell sorter. To ensure that the SORE6 activity in these two cell subsets was stable, we evaluated the GFP expression in the purified SORE6− and SORE6+ derived from SupM2 cells weekly for a total of 7 weeks. As shown in Figure 2B, the GFP expression in SORE6+ cells persisted throughout the 7-week time period and SORE6− cells remained negative for GFP during this time span.

### 3.3. SORE6 Is Sensitive to the Expression Level of Sox2 and Oct4 in ALK + ALCL Cells

To obtain a better appreciation of intra-tumoral heterogeneity in ALK + ALCL cells, we generated single-cell clones derived from purified SORE6− and SORE6+ cells for biological and biochemical studies. All experiments described below were conducted using single-cell clones 5-4 and 5-5 (SORE6−) and single-cell clones 6-5 and 6-6 (SORE6+), all of which were derived from SupM2. To confirm that the SORE6− cells were genuinely negative for SORE6 activity, and that they have sufficient integration of the SORE6 reporter plasmid, we performed PCR using a primer set targeting SORE6. We found approximately equal levels of the amplicons between the SORE6− and SORE6+ single-cell clones, and representative results are illustrated in Figure S1.

**Figure 3:**
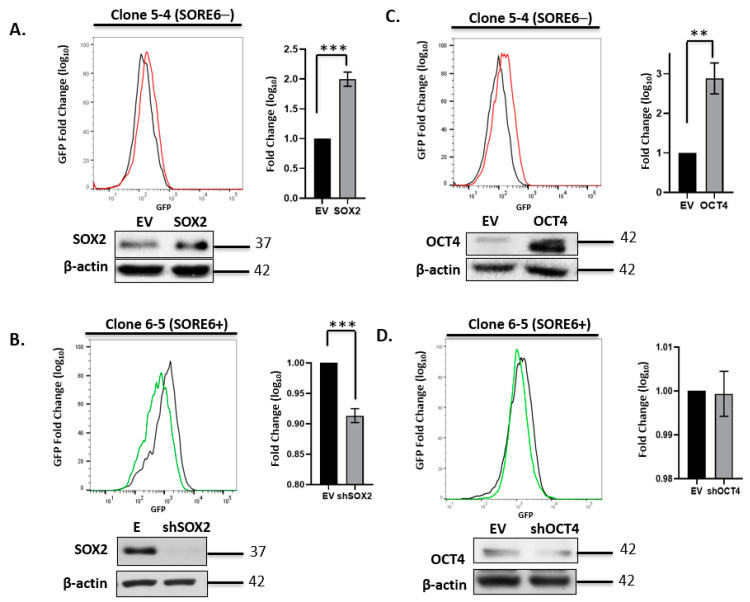
Experimental manipulation of Sox2 and Oct4 in SupM2 SORE6 clones. (**A**) FACS analysis showing the effect on SORE6 reporter activity of overexpressing Sox2 in single-cell clone 5-4 (SupM2 SORE6−). (**B**) FACS analysis showing the effect on SORE6 reporter activity of overexpressing shSox2 in single-cell clone 6-5 (SupM2 SORE6+). (**C**) FACS analysis showing the effect of Oct4 downregulation on SORE6 reporter activity in clone 5-4 (SupM2 SORE6−). (**D**) FACS analysis showing the effect of shOct4 transfection on SORE6 reporter activity in single-cell clone 6-5 (SupM2 SORE6+). All results shown are representative of three independent experiments and were also repeated in a second batch of single-cell clones. Side panels show the fold change of GFP in log scale (mean ± SEM). Bottom panels of each figure are the Western blots showing the effect of transient transfection with Sox2, Oct4, shSox2, and shOct4 plasmids. Empty vector (EV) was included as a negative control. ** *p* < 0.01, *** *p* < 0.001.

We next asked if experimental manipulations of the expression level of Sox2 or Oct4 expression can result in a significant change in the SORE6 reporter activity (i.e., GFP expression level). Triplicate experiments using the four single-cell clones were performed, and representative results are illustrated in Figure 3. There was a relatively small but statistically significant upregulation of GFP expression in SORE6− cells when they were subjected to lentiviral-mediated transduction of a Sox2 expression vector. Lentiviral transduction of shRNA for Sox2 significantly decreased GFP expression in SORE6+ cells, and this change in GFP was more readily appreciated than that induced by Sox2 gene transduction into SORE6− cells. Similarly, gene transduction of an Oct4 expression vector into SORE6− cells resulted in a relatively small but statistically significant increase of GFP. However, the transduction of Oct4 shRNA into SORE6+ cells did not result in any appreciable downregulation of Oct4 or the GFP level. The lack of Oct4 knockdown appeared to be specific to ALK + ALCL cells, since the use of the same shRNA effectively suppressed Oct4 expression in Ntera-2 cells (Figure S2). Overall, in conjunction with several previously published studies using SORE6 in different cell types [20,22,23,24,28], we believe that the SORE6 reporter is a valid measurement of the expression/biological activity of Sox2 and Oct4 in ALK + ALCL cells.

### 3.4. Functional Comparison of SORE6+ and SORE6− Cells

**Figure 4:**
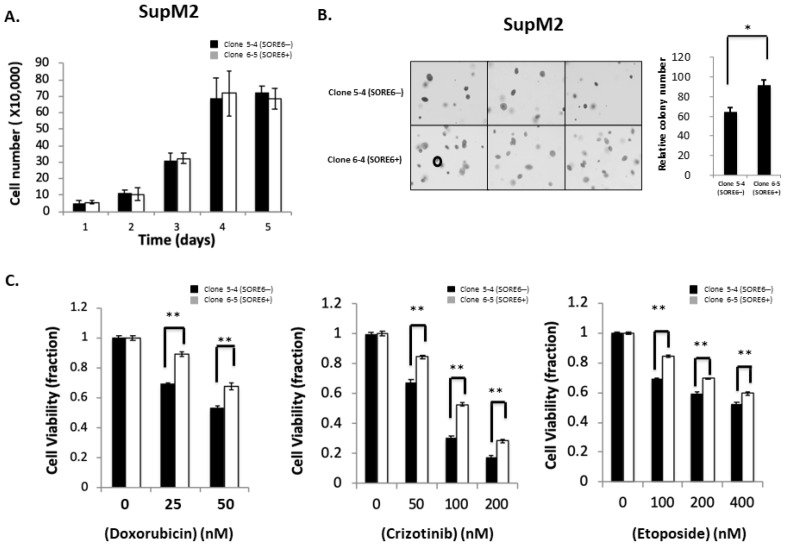
Cell growth, colony formation, and response to therapeutic agents in SORE6− and SORE6+ clones. (**A**) Cell growth of 5-4 (SupM2 SORE6−) and 6-5 (SupM2 SORE6+) clones over the course of 5 days. (**B**) Soft agar colony formation of SORE6− and SORE6+ subsets in SupM2 clones for 10 days. The circle on the bottom-left panel marks the cutoff for a colony to be counted. Triplicate experiments were performed. Experiments were repeated in two single-cell clones. The right panel showed the relative colony numbers in SupM2 cells. Results are mean ± SEM, * *p* < 0.05. (**C**). SupM2 SORE6− and SORE6+ cells after treatment with doxorubicin, crizotinib, and etoposide at the indicated concentrations for 48 h at 5% FBS. Results shown are representative of three independent experiments. ** *p* < 0.01.

We then assessed if there were substantial biological differences between SORE6− and SORE6+ cells. Representative results are illustrated in Figure 4A,B. There was no significant difference in the cell growth between clone 5-4/5-5 and clone 6-5/6-6. However, using the soft agar colony formation assay, we found that the colony counts were significantly higher in SORE6+ compared to SORE6− cells (92 ± 5 versus 61 ± 4; *p* < 0.05). Similar results were obtained when SORE6− and SORE6+ cells derived from UCONN-2 cells were used (5 ± 0.3 versus 10.7 ± 0.5; *p* < 0.05) (Figure S3).

We then examined the chemoresistance properties between the SORE6− cells and SORE6+ cells derived from SupM2. As shown in Figure 4C, SORE6+ clones were significantly more resistant to chemotherapeutic agents, particularly when the concentration of fetal bovine serum added to the tissue culture was lowered to 5%. Specifically, the IC_50_ of doxorubicin in SORE6+ cells was significantly higher than that of SORE6− (67.4 nM versus 52.3 nM; *p* < 0.05). The IC_50_ of crizotinib in SORE6+ cells was significantly higher that of SORE6− cells (130.2 nM versus 67.6 nM; *p* < 0.05). Similarly, the etoposide IC_50_ of SORE6+ cells was significantly higher than that of SORE6− (528.4 nM versus 412.2 nM; *p* < 0.05).

**Figure 5:**
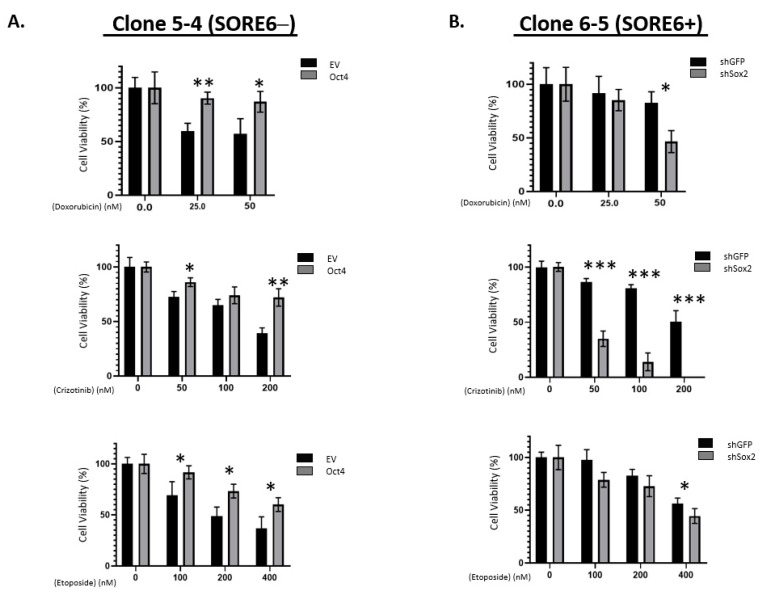
iPS factors enhance chemoresistance of SORE6 clones. (**A**) Overexpression of EV or Oct4 in 5-4 (SupM2 SORE6−) cells, followed by treatment with doxorubicin, crizotinib, and etoposide. (**B**) Overexpression of EV or shSox2 in 6-5 (SupM2 SORE6+) cells, followed by treatment with doxorubicin, crizotinib, and etoposide. Results are mean ± SEM, * *p* < 0.05, ** *p* < 0.01, *** *p* < 0.001.

To assess if Sox2 and Oct4 are directly involved in conferring the phenotypic differences between SORE6− and SORE6+ cells, we manipulated their protein levels and observed how these manipulations resulted in changes in chemoresistance. As shown in Figure 5A, compared to the negative controls, the transduction of Oct4 into SORE6− cells growing in 5% serum significantly increased the IC_50_ value for doxorubicin (281.0 nM versus 49.1 nM), crizotinib (397 nM versus 148.6 nM), and etoposide (623.2 nM versus 212.1 nM). All of these differences are statistically significant. On the other hand, compared to the negative control, shRNA knockdown of Sox2 in SORE6+ cells resulted in their significant sensitization to doxorubicin (60.6 nM versus 227.3 nM), crizotinib (20.5 nM versus 213.6 nM), and etoposide (393 nM versus 721.4 nM) (Figure 5B). All these differences are statistically significant.

### 3.5. SORE6− and SORE6+ Subsets Are Biochemically Distinct

**Figure 6:**
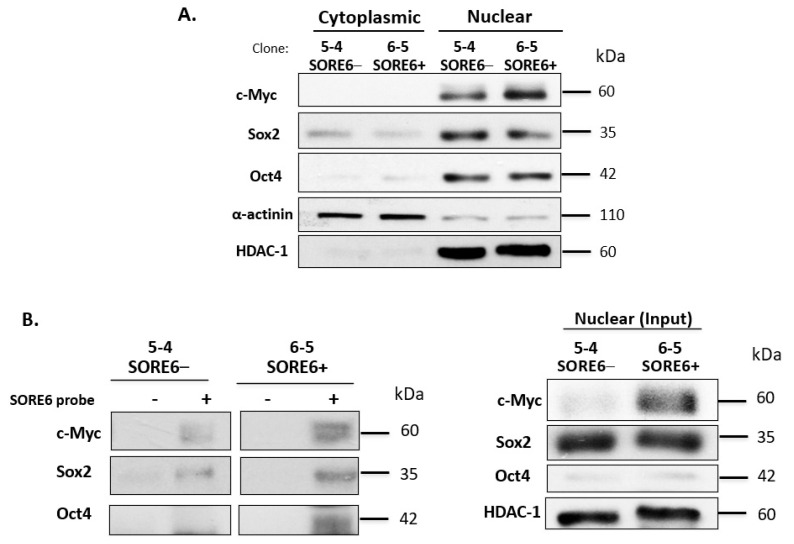
SORE6− and SORE6+ clones are biochemically distinct. (**A**) The subcellular localization of Sox2, Oct4, and c-Myc in SORE6− and SORE6+ cells derived from SupM2, assessed by the nuclear cytoplasmic fractionation assay. (**B**) The DNA pull-down assay was performed to assess Sox2, Oct4, and c-Myc transcriptional activity in SORE6− and SORE6+ cells using a biotin-labeled SORE6 probe.

Next, we performed subcellular fractionation to determine the localization and expression level of Sox2 and Oct4 in SORE6− and SORE6+ cell clones. By cytoplasmic nuclear fractionation, we observed that the protein expression levels of these two proteins and their subcellular localizations were similar between SORE6− and SORE6+ cells. Specifically, both Sox2 and Oct4 were found to be highly concentrated in the nucleus. Since c-Myc was previously found to be a driver of cancer stemness in ALK + ALCL23, we also examined the expression and subcellular localization of this protein. As shown in Figure 6A, c-Myc had substantially higher expression in SORE6+ cells.

To explain the difference in the SORE6 reporter activity between SORE6− and SORE6+ cells, we performed DNA-pull down using a biotin-labeled probe carrying the DNA-binding consensus sequence of SORE6. As shown in Figure 6B, we found more abundant SORE6 binding by Sox2, Oct4, and c-Myc in SORE6+ cells compared to SORE6− cells (Figure 6B). A survey of key cell-signaling proteins by Western blots showed appreciably higher levels of pSTAT3 and pAKT in SORE6+ cells compared to SORE6− cells (Figure S4). No substantial differences were found in pALK. Of note, KLF4—another embryonic stem cell marker—was found to be higher in SORE6+ cells.

### 3.6. SORE6 Is Responsive to the c-Myc Expression Level

**Figure 7:**
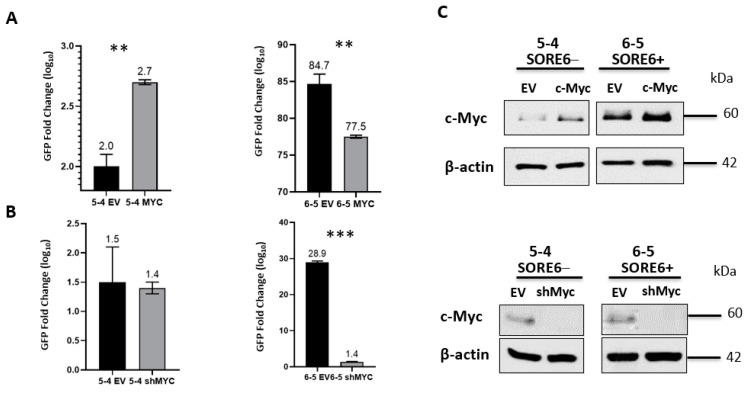
c-Myc regulates the GFP activity of SORE6 clones. (**A**) FACS analysis of GFP activity following overexpression of c-Myc in 5-4 (SupM2 SORE6− clone) and 6-5 (SupM2 SORE6+ clone). (**B**) FACS analysis of GFP activity following downregulation of c-Myc in 5-4 (SupM2 SORE6− clone) and 6-5 (SupM2 SORE6+ clone). (**C**) Western blot indicating successful transfection of c-Myc or shRNA for Myc for both experiments are included in the panels to the right. Results are mean ± SEM, ** *p* < 0.01, *** *p* < 0.001. Of note, the c-Myc band for the SORE6− cells treated with shRNA for c-Myc (Figure 7C, bottom-left corner) was intentionally over-exposed to reveal the otherwise weak c-Myc band.

Since we previously published that the protein expression level of c-Myc is a key regulator of cancer stemness in ALK + ALCL26, we asked if experimental manipulations of c-Myc can modulate the SORE6 reporter activity. As illustrated in Figure 7, the enforced expression of c-Myc significantly increased GFP expression in SORE6− cell clones (*p* < 0.01), whereas shRNA knockdown of c-Myc significantly reduced GFP expression in SORE6+ cells (*p* < 0.001).

## 4. Discussion

Considering the biological and clinical significance of cancer stem cells and CSL cells, extensive research has been done in developing methods that are useful to detect and enrich these unique cell populations. A number of cell-surface proteins and cytoplasmic proteins have been described to be useful markers [16,17,18,19]. Few reporter systems designed to detect the expression and activity of various transcription factors, typically those implicated in the maintenance of pluripotency in embryonic stem cells (e.g., Sox2, Oct4, and c-Myc), have also been described [20,26,29,30,31]. The SORE6 reporter consists of six tandem repeats of a composite Sox2/Oct4 response element derived from the proximal human *NANOG* promoter [20]. Based on a small number of studies using non-hematologic cancer models, SORE6+ cells were found to be enriched with cells with CSL properties [20,23,24,25]. Specifically, SORE6+ cells consistently exhibit higher self-renewal, tumor-initiating capability, resistance to chemotherapeutics, and asymmetric cell division [20]. In this study, we aimed to extend these studies by assessing if the SORE6 reporter is a useful marker to identify and enrich CSL cells in ALK + ALCL, a type of hematologic malignancy.

One of our major aims was to evaluate if the SORE6 reporter is a valid system to identify and enrich CSL cells in ALK + ALCL. In our functional studies, we confirmed that SORE6+ cells are significantly more chemoresistant and clonogenic than SORE6− cells. Our findings support the concept that SORE6+ cells are enriched in CSL properties. As the read-out of this reporter is based on the expression of fluorescence signals, CSL cells can be readily detected and purified using flow cytometry [20]. Furthermore, in our experience, relatively subtle changes in the proportion of CSL cells can be detected, in view of the relatively high sensitivity of flow cytometry. Overall, we conclude that and SORE6 is a useful experimental marker to identify CSL cells.

Sox2 has been implicated to promote invasion, migration, and metastasis in colorectal cancer, glioma, melanoma, gastric cancer, hepatocellular cancer, and ovarian cancer [24,32,33,34,35,36]. In keeping with our prior findings [22], Sox2 was found to be highly expressed in most ALK + ALCL cell lines examined. On the basis of our previous study, the aberrant expression of Sox2 in these lymphoma cells is attributed to the constitutive activation of the NPM-ALK/STAT3 axis, and inhibition of either ALK or STAT3 led to a substantial decrease in Sox2 expression [22]. As shown in this study, the change in the SORE6 activity in SORE6+ cells resulting from Sox2 knockdown was more prominent than that resulting from Sox2 gene transfection in SORE6− cells. This finding is in parallel to that of our previous study in which a Sox2 reporter was used [26]. We believe that these observations are consistent with our model in which the transcriptional activity of Sox2 in ALK + ALCL cells is dependent on a ‘permissive’ environment characterized by a high expression level of c-Myc [26]. Indeed, we found that SORE6+ cells expressed c-Myc to a substantially higher level than SORE6− cells. Thus, the aberrant expression of Sox2 is a necessary but not sufficient condition for this protein to confer cancer stemness. A better understanding of the nature of this ‘permissive’ environment will be of great interest, since one can suppress cancer stemness by modulating this environment. Of interest, we previously found that the transcription activity of Sox2 correlates with its level of phosphorylation in breast cancer cells [37]. It is tempting to speculate that there is a possible link between high c-Myc expression and the activation of tyrosine kinase (s) that can interact with Sox2.

Oct4, also known as Oct3 or Oct3/4, is encoded by the *POU5F1* gene [38]. It is known that Oct4 is overexpressed in many types of cancers [21,39,40,41,42,43], although this protein has not been previously studied in ALK + ALCL. Compared to Ntera-2 cells, the expression level of Oct4 is relatively low in ALK + ALCL cells. We found that the gene transfection of Oct4 into SORE6− cells resulted in a small but detectable increase in SORE6 activity—a pattern similar to that observed in SORE6− cells transfected with Sox2. These findings suggest that a ‘permissive’ environment is also required for Oct4 to exert its transcriptional activity and confer cancer stemness. Perhaps this finding is not too surprising, since Oct4 is known to dimerize with Sox2, and the Oct4–Sox2 heterodimers operate synergistically to regulate the transcription of target genes [44,45,46,47,48,49]. Its expression, albeit in a relatively small quantity, may potentiate the biological effects of Sox2, and the use of the SORE6 reporter holds at least theoretical advantages over the use of the Sox2 reporter. The latter point is particularly relevant when one considers the observation that the stoichiometric ratio of Sox2 and Oct4 has an impact on pluripotency [50]. Thus, it has been reported that increasing the proportion of Sox2 relevant to Oct4 decreases pluripotency, whereas decreasing the proportion of Sox2 shows an opposite effect [50]. Based on our finding that c-Myc is highly expressed in SORE6+ cells, we believe that there will be substantial overlap between cells showing responsiveness to SORE6 and those showing responsiveness to the Sox2 reporter. Whether the SORE6 reporter is truly superior to the Sox2 reporter [26] in enriching CSL is currently under investigation in our laboratory.

Our data support the concept that Sox2 and/or Oct4 is directly responsible for the CSL properties identified in SORE6+ cells. Thus, the transduction of Oct4 into SORE6− cells increased chemoresistance whereas the shRNA knockdown of Sox2 in SORE6+ cells lowered chemoresistance. In further support of the direct role of Sox2/Oct4 in this context, our DNA pull-down experiments showed that the binding by these two proteins to the SORE6 probe was substantially higher in SORE6+ cells. In view of the key roles of Sox2/Oct4 in regulating the pluripotency of embryonic stem cells, it is tempting to speculate that Sox2/Oct4 leverages the same mechanisms and cellular pathways as in embryonic stem cells. Of note, the NPM-ALK/STAT3 status was found to be slightly different between SORE6− and SORE6+ cells, and it is likely that these two key oncogenic proteins in ALK + ALCL do not play a significant role in generating the SORE6−/SORE6+ dichotomy. On the other hand, modulation of the c-Myc expression level can significantly change the SORE6 activity. Additionally, abundant interaction between c-Myc and the SORE6 DNA probe was found in SORE6+ but not SORE6− cells. It remains to be determined whether the binding between c-Myc and SORE6 is direct or dependent on other proteins such as Sox2.

## 5. Conclusions

SORE6 is useful in identifying/enriching CSL cells in ALK + ALCL. This reporter can provide a versatile study model to delineate the mechanisms underlying cancer stemness and plasticity, which are believed to be key contributing factors to cancer relapses.

## Data Availability

Not applicable.

## Supplementary Materials

The following are available online at https://www.mdpi.com/1467-3045/43/2/41/s1. Figure S1: Confirmation of SORE6 reporter plasmid SupM2 SORE6 clone 5-4 (SORE6−) and 6-5 (SORE6+). Figure S2: Cell growth and colony formation ability of UCONN-L2 SORE6− and SORE6+ clones. Figure S3: Analysis of total and phosphorylated proteins in 5-4 (SupM2 SORE6−) and 6-5 (SORE6+) clones.

## References

[1] Fornari A., Piva R., Chiarle R., Novero D., Inghirami G. (2009). Anaplastic large cell lymphoma: One or more entities among T-cell lymphoma?. Hematol. Oncol..

[2] Amin H.M., Lai R. (2007). Pathobiology of ALK+ anaplastic large-cell lymphoma. Blood.

[3] Kinney M.C., Higgins R.A., Medina E.A. (2011). Anaplastic large cell lymphoma: Twenty-five years of discovery. Arch. Pathol. Lab. Med..

[4] Delsol G. (2008). Anaplastic Large Cell Lymphoma (ALCL), ALK-Positive, International Agency for Research on Cancer (IARC).

[5] Morris S.W., Kirstein M.N., Valentine M.B., Dittmer K.G., Shapiro D.N., Saltman D.L., Look A.T. (1994). Fusion of a kinase gene, ALK, to a nucleolar protein gene, NPM, in non-Hodgkin’s lymphoma. Science.

[6] Bastard C., Rimokh R., Dastugue N., Huret J.-L., Kristoffersson U., Magaud J.-P., Nezelof C., Tilly H., Vannier J.-P., Hemet J. (1990). CD30-positive large cell lymphomas (‘Ki-1 lymphoma’) are associated with a chromosomal translocation involving 5q35. Br. J. Haematol..

[7] Choudhari R., Minero V.G., Menotti M., Pulito R., Brakebusch C., Compagno M., Voena C., Ambrogio C., Chiarle R. (2016). Redundant and nonredundant roles for Cdc42 and Rac1 in lymphomas developed in NPM-ALK transgenic mice. Blood.

[8] Moti N., Malcolm T., Hamoudi R., Mian S.H., Garland G., Hook C.E., Burke G.A.A., Wasik M.A., Merkel O., Kenner L. (2015). Anaplastic large cell lymphoma-propagating cells are detectable by side population analysis and possess an expression profile reflective of a primitive origin. Oncogene.

[9] Chihara D., Fanale M.A., Miranda R.N., Noorani M., Westin J.R., Nastoupil L.J., Hagemeister F.B., Fayad L.E., Romaguera J.E., Samaniego F. (2017). The survival outcome of patients with relapsed/refractory peripheral T-cell lymphoma-not otherwise specified and angioimmunoblastic T-cell lymphoma. Br. J. Haematol..

[10] Zinzani P.L. (2017). ALCL: Is it now a curable disease?. Blood.

[11] Meacham C.E., Morrison S.J. (2013). Tumour heterogeneity and cancer cell plasticity. Nat. Cell Biol..

[12] Marusyk A., Almendro V., Polyak K. (2012). Intra-tumour heterogeneity: A looking glass for cancer?. Nat. Rev. Cancer..

[13] Costello R., Mallet F., Gaugler B., Sainty D., Arnoulet C., Gastaut J.A., Olive D. (2000). Human acute myeloid leukemia CD34+/CD38- progenitor cells have decreased sensitivity to chemotherapy and Fas-induced apoptosis, reduced immunogenicity, and impaired dendritic cell transformation capacities. Cancer Res..

[14] Dean M., Fojo T., Bates S.E. (2005). Tumour stem cells and drug resistance. Nat. Rev. Cancer.

[15] Guzman M.L., Swiderski C.F., Howard D.S., Grimes B.A., Rossi R.M., Szilvassy S.J., Jordan C.T. (2002). Preferential induction of apoptosis for primary human leukemic stem cells. Proc. Natl. Acad. Sci. USA.

[16] Klonisch T., Wiechec E., Hombach-Klonisch S., Ande S.R., Wesselborg S., Schulze-Osthoff K., Los M. (2008). Cancer stem cell markers in common cancers—Therapeutic implications. Trends Mol. Med..

[17] D’Angelo R.C., Wicha M.S. (2010). Stem Cells in Normal Development and Cancer. Prog. Mol. Biol. Transl. Sci..

[18] Magee J.A., Piskounova E., Morrison S.J. (2012). Cancer Stem Cells: Impact, Heterogeneity, and Uncertainty. Cancer Cell.

[19] Visvader J.E., Lindeman G. (2012). Cancer Stem Cells: Current Status and Evolving Complexities. Cell Stem Cell.

[20] Tang B., Raviv A., Esposito D., Flanders K.C., Daniel C., Nghiem B.T., Garfield S., Lim L., Mannan P., Robles A. (2015). A Flexible Reporter System for Direct Observation and Isolation of Cancer Stem Cells. Stem Cell Rep..

[21] Schoenhals M., Kassambara A., De Vos J., Hose D., Moreaux J., Klein B. (2009). Embryonic stem cell markers expression in cancers. Biochem. Biophys. Res. Commun..

[22] Gelebart P., Hegazy S.A., Wang P., Bone K.M., Anand M., Sharon D., Hitt M., Pearson J.D., Ingham R.J., Ma Y. (2012). Aberrant expression and biological significance of Sox2, an embryonic stem cell transcriptional factor, in ALK-positive anaplastic large cell lymphoma. Blood Cancer J..

[23] Menendez S.T., Rey V., Martinez-Cruzado L., Gonzalez M.V., Morales-Molina A., Santos L., Blanco V., Alvarez C., Estupiñan O., Allonca E. (2020). SOX2 Expression and Transcriptional Activity Identifies a Subpopulation of Cancer Stem Cells in Sarcoma with Prognostic Implications. Cancers.

[24] Pádua D., Barros R., Amaral A.L., Mesquita P., Freire A.F., Sousa M., Maia A.F., Caiado I., Fernandes H., Pombinho A. (2020). A SOX2 Reporter System Identifies Gastric Cancer Stem-Like Cells Sensitive to Monensin. Cancers.

[25] Saygin C., Samour M., Chumakova A., Jarrar A., Lathia J.D., Reizes O. (2016). Reporter Systems to Study Cancer Stem Cells. Methods Mol. Biol..

[26] Wu C., Zhang H.F., Gupta N., Alshareef A., Wang Q., Huang Y., Lewis J.T., Douglas D.N., Kneteman N.M., Lai R. (2016). A positive feedback loop involving the Wnt/beta-catenin/MYC/Sox2 axis defines a highly tumorigenic cell subpopulation in ALK-positive anaplastic large cell lymphoma. J. Hematol. Oncol..

[27] Lee V.M., Andrews P.W. (1986). Differentiation of NTERA-2 clonal human embryonal carcinoma cells into neurons involves the induction of all three neurofilament proteins. J. Neurosci..

[28] Blum W., Pecze L., Felley-Bosco E., Wu L., De Perrot M., Schwaller B. (2017). Stem Cell Factor-Based Identification and Functional Properties of In Vitro-Selected Subpopulations of Malignant Mesothelioma Cells. Stem Cell Rep..

[29] Mohammed M.K., Shao C., Wang J., Wei Q., Wang X., Collier Z., Tang S., Liu H., Zhang F., Huang J. (2016). Wnt/beta-catenin signaling plays an ever-expanding role in stem cell self-renewal, tumorigenesis and cancer chemoresistance. Genes Dis..

[30] Pan G., Thomson J.A. (2007). Nanog and transcriptional networks in embryonic stem cell pluripotency. Cell Res..

[31] Wang Z., Li Y., Banerjee S., Sarkar F.H. (2008). Exploitation of the Notch signaling pathway as a novel target for cancer therapy. Anticancer. Res..

[32] Han X., Fang X., Lou X., Hua D., Ding W., Foltz G., Hood L., Yuan Y., Lin B. (2012). Silencing SOX2 Induced Mesenchymal-Epithelial Transition and Its Expression Predicts Liver and Lymph Node Metastasis of CRC Patients. PLoS ONE.

[33] Alonso M.M., Diez-Valle R., Manterola L., Rubio A., Liu D., Cortes-Santiago N., Urquiza L., Jauregi P., De Munain A.L., Sampron N. (2011). Genetic and Epigenetic Modifications of Sox2 Contribute to the Invasive Phenotype of Malignant Gliomas. PLoS ONE.

[34] Girouard S.D., Laga A.C., Mihm M.C., Scolyer R.A., Thompson J., Zhan Q., Widlund H., Lee C.-W., Murphy G.F. (2012). SOX2 contributes to melanoma cell invasion. Lab. Investig..

[35] Sun C., Sun L., Li Y., Kang X., Zhang S., Liu Y. (2013). Sox2 expression predicts poor survival of hepatocellular carcinoma patients and it promotes liver cancer cell invasion by activating Slug. Med. Oncol..

[36] Lou X., Han X., Jin C., Tian W., Yu W., Ding D., Cheng L., Huang B., Jiang H., Lin B. (2013). SOX2 Targets Fibronectin 1 to Promote Cell Migration and Invasion in Ovarian Cancer: New Molecular Leads for Therapeutic Intervention. OMICS A J. Integr. Biol..

[37] Gupta N., Gopal K., Wu C., Alshareef A., Chow A., Wu F., Wang P., Ye X., Bigras G., Lai R. (2018). Phosphorylation of Sox2 at Threonine 116 is a Potential Marker to Identify a Subset of Breast Cancer Cells with High Tumorigenecity and Stem-Like Features. Cancers.

[38] Nichols J., Zevnik B., Anastassiadis K., Niwa H., Klewe-Nebenius D., Chambers I., Schöler H., Smith A. (1998). Formation of Pluripotent Stem Cells in the Mammalian Embryo Depends on the POU Transcription Factor Oct4. Cell.

[39] Chiou S.-H., Wang M.-L., Chou Y.-T., Chen C.-J., Hong C.-F., Hsieh W.-J., Chang H.-T., Chen Y.-S., Lin T.-W., Hsu H.-S. (2010). Coexpression of Oct4 and Nanog enhances malignancy in lung adenocarcinoma by inducing cancer stem cell-like properties and epithelial-mesenchymal transdifferentiation. Cancer Res..

[40] Meng H.-M., Zheng P., Wang X.-Y., Liu C., Sui H.-M., Wu S.-J., Zhou J., Ding Y.-Q., Li J. (2010). Over-expression of Nanog predicts tumor progression and poor prognosis in colorectal cancer. Cancer Biol. Ther..

[41] Wen J., Park J.Y., Park K.H., Chung H.W., Bang S., Park S.W., Song S.Y. (2010). Oct4 and Nanog Expression Is Associated With Early Stages of Pancreatic Carcinogenesis. Pancreas.

[42] Kim B.W., Cho H., Choi C.H., Ylaya K., Chung J.-Y., Kim J.-H., Hewitt S.M. (2015). Clinical significance of OCT4 and SOX2 protein expression in cervical cancer. BMC Cancer.

[43] Guzel E., Karatas O.F., Duz M.B., Solak M., Ittmann M., Ozen M. (2014). Differential expression of stem cell markers and ABCG2 in recurrent prostate cancer. Prostate.

[44] Chen X., Xu H., Yuan P., Fang F., Huss M., Vega V.B., Wong E., Orlov Y., Zhang W., Jiang J. (2008). Integration of External Signaling Pathways with the Core Transcriptional Network in Embryonic Stem Cells. Cell.

[45] Saijoh Y., Fujii H., Meno C., Sato M., Hirota Y., Nagamatsu S., Lkeda M., Hamada H. (1996). Identification of putative downstream genes of Oct-3, a pluripotent cell-specific transcription factor. Genes Cells.

[46] Yuan H., Corbi N., Basilico C., Dailey L. (1995). Developmental-specific activity of the FGF-4 enhancer requires the synergistic action of Sox2 and Oct-3. Genes Dev..

[47] Nishimoto M., Fukushima A., Okuda A., Muramatsu M. (1999). The gene for the embryonic stem cell coactivator UTF1 carries a regulatory element which selectively interacts with a complex composed of Oct-3/4 and Sox-2. Mol. Cell Biol..

[48] Tomioka M., Nishimoto M., Miyagi S., Katayanagi T., Fukui N., Niwa H., Muramatsu M., Okuda A. (2002). Identification of Sox-2 regulatory region which is under the control of Oct-3/4-Sox-2 complex. Nucleic Acids Res..

[49] Rizzino A. (2013). Concise review: The Sox2-Oct4 connection: Critical players in a much larger interdependent network integrated at multiple levels. Stem Cells.

[50] Papapetrou E.P., Tomishima M.J., Chambers S.M., Mica Y., Reed E., Menon J., Tabar V., Mo Q., Studer L., Sadelain M. (2009). Stoichiometric and temporal requirements of Oct4, Sox2, Klf4, and c-Myc expression for efficient human iPSC induction and differentiation. Proc. Natl. Acad. Sci. USA.

