# Correction: Li et al. Identification and Characterization of Cancer Stem-Like Cells in ALK-Positive Anaplastic Large Cell Lymphoma Using the SORE6 Reporter. *Curr. Issues Mol. Biol.* 2021, *43*, 543–557

**DOI:** 10.3390/cimb44100346

**Published:** 2022-10-21

**Authors:** Jing Li, Moinul Haque, Chuquan Shang, Bardes Hassan, Dongzhe Liu, Will Chen, Raymond Lai

**Affiliations:** 1 Department of Laboratory Medicine and Pathology, University of Alberta, Edmonton, AB T6G 2E1, Canada; 2 Electron Microscopy Center, Basic Medical Science College, Harbin Medical University, Harbin 150080, China; 3 College of Medicine and Health, University College Cork, T12 AK54 Cork, Ireland; 4 Department of Pathology, Faculty of Veterinary Medicine, Cairo University, Giza 12211, Egypt; 5 Laboratory of Biology and Chemistry, Basic Medical Science College, Harbin Medical University, Harbin 150080, China; 6 Department of Oncology, University of Alberta, Edmonton, AB T6G 2R7, Canada

## Error in Figure

In the original publication [1], there was a mistake in Figure 4 as published. The lower panel of Figure 4B should have displayed three distinct soft agar replicates for Clone 6–4 (SORE6+). A formatting error in this panel resulted in two copies of the first replicate being shown. The corrected Figure 4 appears below with all distinct replicates.

**Figure 4:**
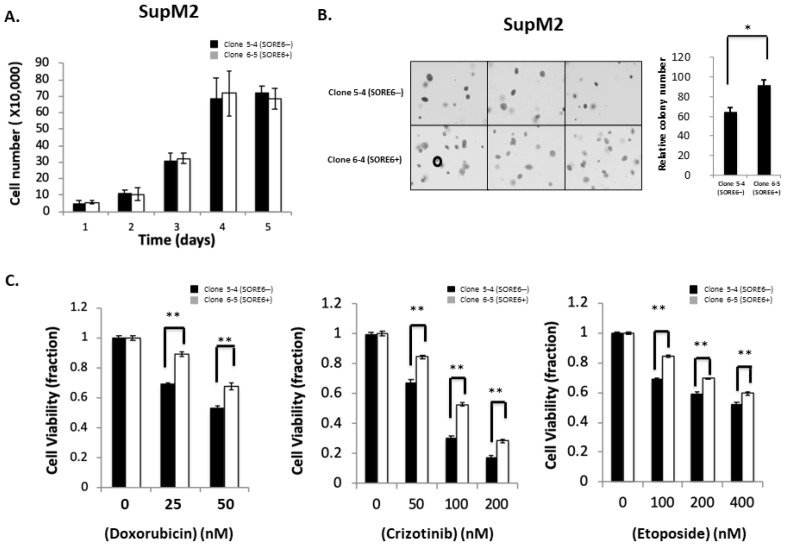
Cell growth, colony formation, and response to therapeutic agents in SORE6− and SORE6+clones. (**A**) Cell growth of 5-4 (SupM2 SORE6−) and 6-5 (SupM2 SORE6+) clones over the course of 5 days. (**B**) Soft agar colony formation of SORE6− and SORE6+ subsets in SupM2 clones for 10 days. The circle on the bottom-left panel marks the cutoff for a colony to be counted. Triplicate experiments were performed. Experiments were repeated in two single-cell clones. The right panel showed the relative colony numbers in SupM2 cells. Results are mean ± SEM, * *p* < 0.05. (**C**). SupM2 SORE6− and SORE6+ cells after treatment with doxorubicin, crizotinib, and etoposide at the indicated concentrations for 48 h at 5% FBS. Results shown are representative of three independent experiments. ** *p* < 0.01.

The authors state that the scientific conclusions are unaffected. This correction was approved by the Academic Editor. The original publication has also been updated.
