# Supplementary material for: Identification and Characterization of Cancer Stem-Like Cells in ALK-Positive Anaplastic Large Cell Lymphoma Using the SORE6 Reporter
Source: Curr Issues Mol Biol. 2021 Jul 2;43(2):0. doi: 10.3390/cimb43020041 (PMC8929104; doi:10.3390/cimb43020041)
Supplement: Supplementary file 1 [file cimb-43-00041-s001.zip › cimb-1214647-supplementary.pdf]

Supplementary Materials

Identification and Characterization of Cancer Stem-like Cells in ALK-Positive Anaplastic Large Cell Lymphoma Using the SORE6 Reporter

Jing Li [1,2¶], Moinul Haque [1,3¶], Chuquan Shang [1], Bardes Hassan [1,4], Dongzhe Liu [1,5,6], Will Chen [1] and Raymond Lai[1,7]

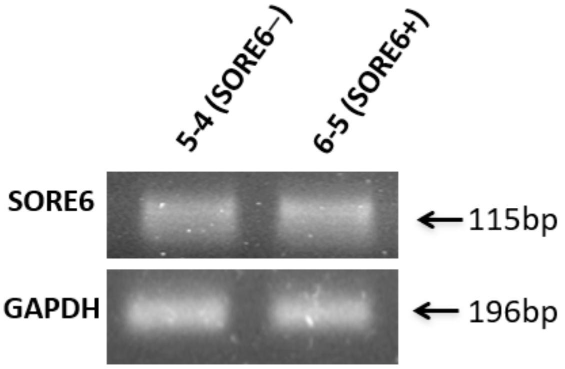

Figure S1. Confirmation of SORE6 reporter plasmid SupM2 SORE6 clone 5-4 (SORE6–) and 6-5 (SORE6+). PCR amplification was performed to identify presence of SORE region in SORE6– and SORE6+ cells. GAPDH was used as endogenous control.

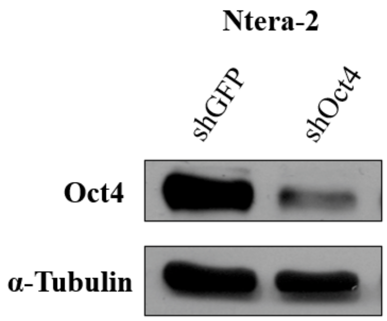

Figure S2. Knockdown of Oct4 in Ntera-2 cells using shRNA. The same Oct4 shRNA was used in all experiments in the main manuscript.

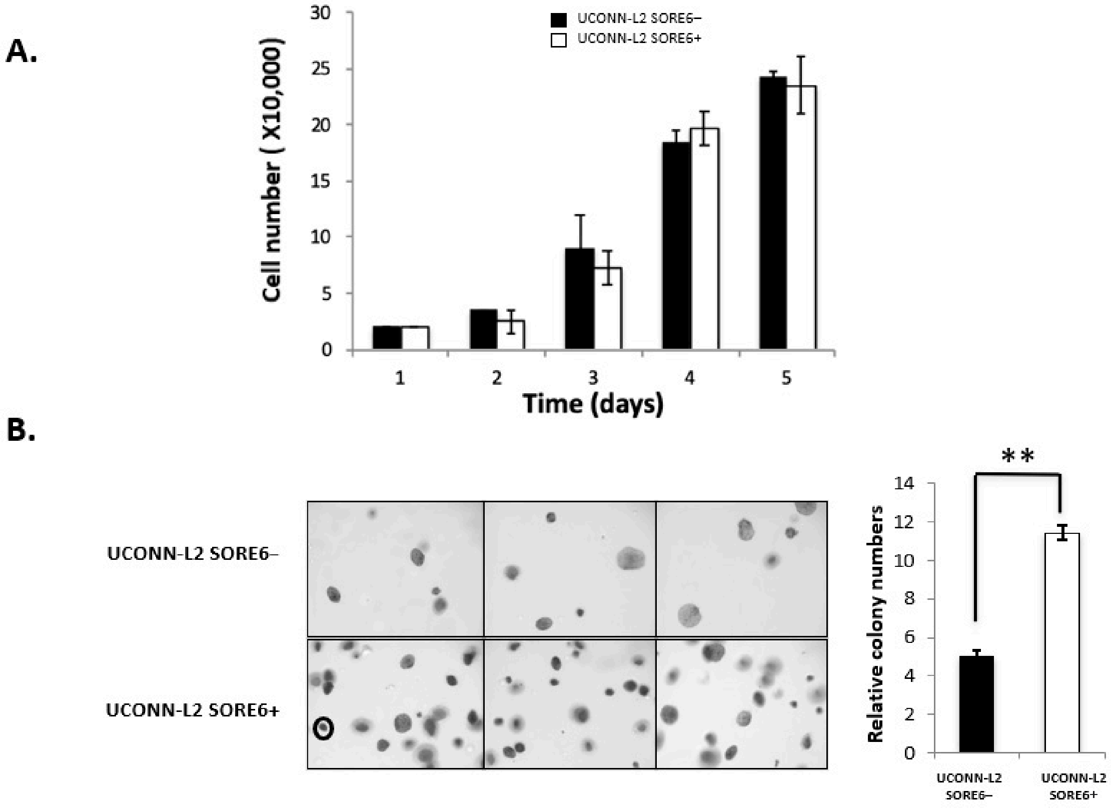

Figure S3. Cell growth and colony-formation ability of UCONN-L2 SORE6– and SORE6+ clones. (**A**) Cell growth of UCONN-L2 SORE6– and SORE6+ clones over the course of 5 days. (**B**) Soft-agar assay of UCONN-L2 SORE6– and SORE6+ clones over the course of 10 days. The black circle on the lower left panel indicates the size cut-off for a colony.

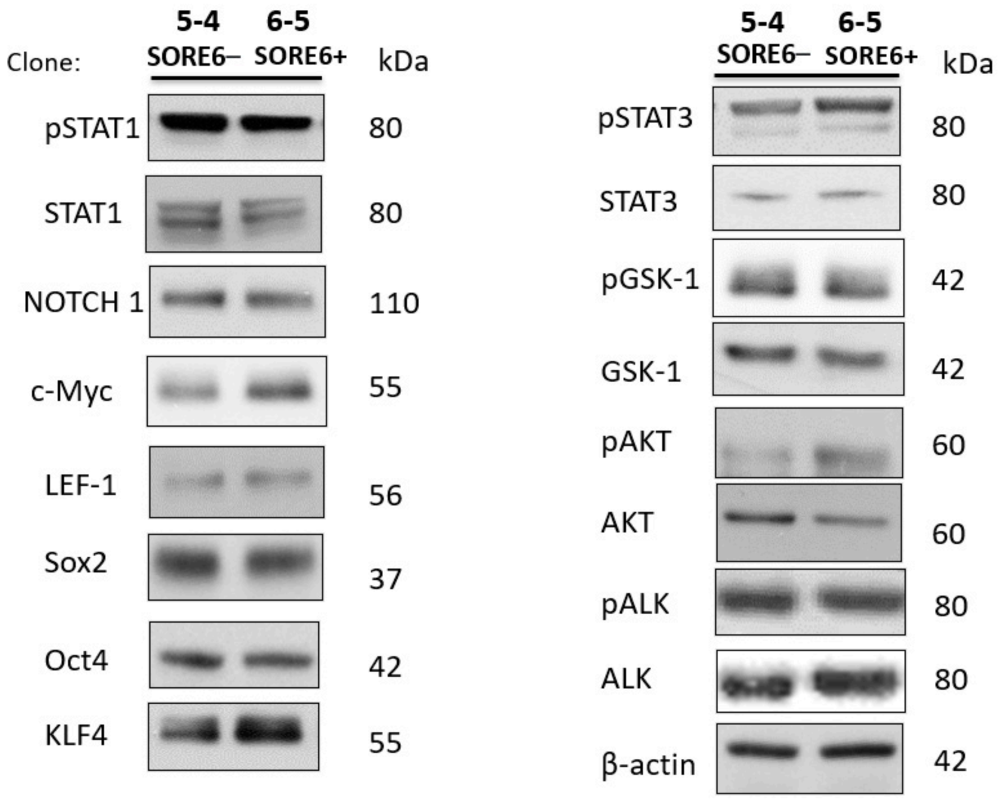

Figure S4. Analysis of total and phosphorylated proteins in 5-4 (SupM2 SORE6–) and 6-5 (SORE6+) clones. Western blotting of a panel of total and phosphorylated proteins was performed in SORE6– and SORE6+ clones.
